# Prediction of Cardiorespiratory Fitness by the Six-Minute Step Test and Its Association with Muscle Strength and Power in Sedentary Obese and Lean Young Women: A Cross-Sectional Study

**DOI:** 10.1371/journal.pone.0145960

**Published:** 2015-12-30

**Authors:** Lívia Pinheiro Carvalho, Luciana Di Thommazo-Luporini, Mylène Aubertin-Leheudre, José Carlos Bonjorno Junior, Cláudio Ricardo de Oliveira, Rafael Luís Luporini, Renata Gonçalves Mendes, Katiany Thais Lopes Zangrando, Renata Trimer, Ross Arena, Audrey Borghi-Silva

**Affiliations:** 1 Cardiopulmonary Physiotherapy Laboratory, Physiotherapy Department, Federal University of Sao Carlos, Sao Carlos, Sao Paulo, Brazil; 2 Kinanthropology Department, University of Quebec in Montreal, Montreal, Quebec, Canada; 3 Medicine Department, Federal University of Sao Carlos, Sao Carlos, Sao Paulo, Brazil; 4 Santa Casa de Misericórdia de Sao Carlos, São Carlos, SP, Brazil; 5 Integrative Physiology Laboratory, Physical Therapy Department, College of Applied Health Sciences, University of Illinois Chicago, Chicago, Illinois, United States of America; Louisiana State University, UNITED STATES

## Abstract

Impaired cardiorespiratory fitness (CRF) is a hallmark characteristic in obese and lean sedentary young women. Peak oxygen consumption (VO_2peak_) prediction from the six-minute step test (6MST) has not been established for sedentary females. It is recognized that lower-limb muscle strength and power play a key role during functional activities. The aim of this study was to investigate cardiorespiratory responses during the 6MST and CPX and to develop a predictive equation to estimate VO_2peak_ in both lean and obese subjects. Additionally we aim to investigate how muscle function impacts functional performance. Lean (LN = 13) and obese (OB = 18) women, aged 20–45, underwent a CPX, two 6MSTs, and isokinetic and isometric knee extensor strength and power evaluations. Regression analysis assessed the ability to predict VO_2peak_ from the 6MST, age and body mass index (BMI). CPX and 6MST main outcomes were compared between LN and OB and correlated with strength and power variables. CRF, functional capacity, and muscle strength and power were lower in the OB compared to LN (<0.05). During the 6MST, LN and OB reached ~90% of predicted maximal heart rate and ~80% of the VO_2peak_ obtained during CPX. BMI, age and number of step cycles (NSC) explained 83% of the total variance in VO_2peak_. Moderate to strong correlations between VO_2peak_ at CPX and VO_2peak_ at 6MST (r = 0.86), VO_2peak_ at CPX and NSC (r = 0.80), as well as between VO_2peak_, NSC and muscle strength and power variables were found (p<0.05). These findings indicate the 6MST, BMI and age accurately predict VO_2peak_ in both lean and obese young sedentary women. Muscle strength and power were related to measures of aerobic and functional performance.

## Introduction

Obesity is a widespread and growing problem worldwide and it is one of the major current public health issues. Impaired cardiorespiratory fitness (CRF) and a sedentary lifestyle are typical characteristics, not only in obese people, but also in normal-weight young adults, especially in women [1,2]. As an important component of prevention and treatment in obesity and physical deconditioning, assessing CRF and prescribing an individualized exercise program is essential [3], as is the identification of cardiovascular and musculoskeletal abnormalities that may impact the exercise performance.

Cardiopulmonary exercise testing (CPX) is useful to objectively assess the integrated physiological systems response to dynamic exercise and is considered the gold standard method to evaluate aerobic performance [3,4]. However, because of its high technical and cost requirements, indirect protocols for peak oxygen consumption (VO_2peak_) prediction have been developed to minimize these barriers in clinical settings [5–11]. Among the most common functional test protocols, the step test stands out for its low cost, simplicity, portability and accessibility [12–15].

At this point, many questions regarding CRF, functional capacity and muscle function, and their relationships, remain unanswered in both obese and non-obese individuals. For example, lower-limb muscle strength and power are purported to play a key role during functional daily activities and have been associated with gait speed, stair climbing ability, and getting up from a seated position [16–18]. Thus, in the last few years, increasing attention has been devoted to the study of muscle force-generation ability in the presence or absence of obesity [19–25], although literature regarding its behavior as well as its involvement in functional performance and CRF is still lacking.

Therefore, the present study aims to evaluate and correlate the cardiorespiratory responses during the 6MST and the CPX, in order to investigate the intensity of this functional test in contrast to the gold standard CRF assessment and, additionally, to develop a predictive equation to estimate VO_2peak_ in both lean and obese sedentary young women. Furthermore, we aim to investigate to what extent muscle strength and power influence functional performance.

We hypothesize that the 6MST predicts maximal cardiorespiratory responses and its estimation would be applicable in both lean and obese young women. Moreover, its performance would be associated with lower-limb muscle strength and power. In addition, other baseline individual characteristics would enhance accuracy of the predictive equation.

## Materials and Methods

### Design and study population

Sedentary lean and obese women aged 20 to 45 years old were recruited from the community via social communication within the department and university, community journals, as well as radio and television media advertisement. After potential subjects responded to the first mail or telephone screening, which took into account the main inclusion and exclusion criteria, they were submitted to the first day of evaluation. Qualified volunteers were allocated into a lean group (LN, n = 13) or obese group (OB, n = 18) according to their Body Mass Index (BMI) and body fat mass (BF): 1) LN = <25 kg.m^-2^ and <35% body fat mass (BF); and 2) OB = ≥35 kg.m^-2^ and ≥40% BF. The OB contained severe and morbidly obese women exclusively. Additionally, only individuals who maintained a stable body mass for at least 6 months without having done a nutritional intervention or any type of aerobic or anaerobic supervised exercise program were included in the study. The criteria for a sedentary lifestyle was <150 minutes per week of structured low-level physical activity. The exclusion criteria considered were: pregnancy, premature menopause, gynecological and/ or orthopedic surgeries, tobacco use and/or abstinence period less than one year before study initiation, drinking alcohol more than twice a week, use of medications, diagnosis of pulmonary, cardiovascular and metabolic diseases, neurological disorders, difficulty in understanding experimental procedures or cognitive impairment.

The study procedures were performed on non-consecutive days separated by at least 48 hours: 1) *1* ^*st*^ *Day*: clinical evaluation was comprised of anamnesis, anthropometric measures, body composition analysis, Baecke physical activity questionnaire, spirometry to evaluate basal pulmonary function, and familiarization with ergometers and testing procedures; 2) *2* ^*nd*^ *Day*: Blood collection and analysis; 3) *3* ^*rd*^ *Day*: treadmill CPX; 4) *4* ^*th*^ *Day*: First 6MST; 5) *5* ^*th*^ *Day*: Second 6MST; and 6) *6* ^*th*^ *Day*: Isometric and isokinetic strength measures. The authors conducted the study after the Ethics Committee of Federal University of São Carlos (CEP-UFSCar: opinion N. 326.607) approval for human research and all volunteers agreed and signed a written informed consent.

### 1^st^ Day

A first clinical anamnesis was taken in an interview format. Besides clinical and anthropometrics evaluations, the participants answered the validated Baecke physical activity questionnaire [26], which includes questions about perceptual physical effort at work, leisure time and sport activities. This procedure enabled evaluators to assess sedentary lifestyle.

Height and body mass (BM) were measured (Welmy 104-A, Santa Bárbara do Oeste, SP, Brazil) in a standing position with bodies stretched upward to the fullest extension, heals to the wall, looking forward and without shoes and shirts. Weight as well as other body composition variables were obtained from tetrapolar bioelectrical impedance analysis, assessed by a Tanita body composition analyzer (Model BC-558, Ironmann, Tanita Corporation, Tokyo, Japan). Assessment procedures followed manufacturer guidelines. All volunteers attended the evaluation at the follicular phase of the menstrual cycle to avoid a hormonal effects bias. Subjects were instructed to have a 4-hour absolute fast, to wear light weight clothes, not have metal in contact with their bodies and to urinate prior to the examination. Appendicular lean mass of the dominant leg (LLM) and appendicular skeletal muscle mass index (ASMI) [27], BF, and total body lean mass (LM) were obtained.

Thigh, waist, and hip circumferences were measured to the nearest 1 cm in accordance with WHO standards [28]. Three measurements were taken from each marked location and the mean of 2 values differing less than 10% was used for statistical analysis.

Pulmonary function was investigated by means of the forced and slow maneuvers (Oxycon Mobile^®^, Mijnhardt/Jäger, Würzburg, German) according to the standard guidelines and Brazilian predicted values [29] for spirometry, ensuring inclusion of volunteers with normal pulmonary function.

### 2^nd^ Day

For blood analysis, the volunteers were instructed to fast 12 to 14 hours, and the collection was carried out between 7:30 and 8:30 in the morning. The volunteers were instructed not to exercise within 48 hours of the exam and to maintain their usual diet.

Blood samples were collected from the upper limb antecubital vein by a qualified professional in a specialized laboratory with certification and quality control by the National Society of Clinical Pathology/ Laboratorial Medicine, recognized by the Brazil Ministry of Health via the National Health Surveillance Agency. Levels of blood fasting glucose and insulin and subsequent insulin resistance index by the Homeostasis Model Assessment method (HOMA-IR) and lipid profile (total cholesterol, HDL-C, LDL-C, VLDL-C and triglycerides) were quantified.

### Exercise Testing and Strength Protocols

All exercise testing and experimental procedures were performed at the same period of the day because of the influence of circadian rhythm, in a standard acclimatized room with a 22–24°C temperature and moderate relative air humidity (40–60%). For exercise testing, subjects were instructed to abstain from alcoholic or stimulant beverages or foods, to not perform physical activities during the previous 48 hours, to not attend the evaluation fasting for more than 2 hours and to report any acute pain or inflammatory processes.

### 3^rd^ Day: Cardiopulmonary Exercise Testing (CPX)

A symptom-limited CPX was conducted on a treadmill (Master Super ATL, Inbramed/Inbrasport, Porto Alegre, RS, Brazil) by one physician and two physical therapists according to the Bruce protocol; the procedures and interruption criteria followed American Thoracic Society recommendations [3]. CPX consisted of a four-minute rest in a sitting and standing position before exercise testing initiation and a three-minute monitored recovery period.

All subjects met at least the following criteria: 1) maximal respiratory exchange ratio (RER) > 1.15; and 2) maximal HR >90% of the maximum age-predicted for women (i.e., 220-age). All measures were collected breath-by-breath using a portable metabolic system (Oxycon Mobile^®^, Mijnhardt/Jäger, Würzburg, German) by means of a mask interface. VO_2peak_, the gold-standard measure of CRF, was defined as the highest 15-second averaged VO_2_ value. Besides VO_2peak_, other ventilatory and metabolic data from CPX as well as those from the functional step test were transferred to a spreadsheet program (Microsoft Excel) for further analysis (4)

A 12-lead electrocardiogram (Wincardio System, Micromed, Brasília, Brazil), heart rate (HR), noninvasive blood pressure (BP), and perceived exertion by the CR-10 Borg scale [30] were also measured and registered.

### 4^th^ and 5^th^ Days: Test-retest Six-minute Step Test (6MST)

Two cadence-free and time-limited step tests were performed on a 15 cm-high single-step platform on different days with a minimum interval of 48 hours between them. In each minute of the test, volunteers received the same standardized encouragement according to the ATS/American College of Chest Physicians recommendations applied for the six-minute walking test [31]. They were encouraged to go up and down the step as many times as they could over 6 minutes and they were instructed to start with their dominant leg. They were allowed to interrupt and return to exercising as well as reduce or increase speed according to their effort perception.

The number of step cycles (NSC) was registered by two trained observers in its totality and partial every-minute step cycles. The same metabolic system for analysis of ventilatory expired gases as well as the method for peak value determination during CPX were used for the 6MST. The workload performed during the test was calculated according to a previous study [32]. Heart rate and BP were measured at rest, at peak exercise, and during the recovery period as well as the CR-10 Borg scale throughout the 6MST protocol.

### 6^th^ Day: Isometric and Isokinetic Strength Measures

Evaluations of the concentric and isometric extensor knee torques were carried out using an isokinetic dynamometer (Biodex Multi-Joint System 3; Biodex Medical System, Inc., Shirley, NY) from at least 48 hours after the last functional test. A concentric evaluation was chosen essentially because of the shortening contraction of knee extensors during the act of going up steps or stairs. Thus, a concentric strength evaluation of these muscles might be more closely related to lower limb functional capacity in this daily activity.

The dynamometer was calibrated every day of testing according to the manufacturer specifications. Isometric and isokinetic assessments were performed using a protocol similar to that used in previous studies [21,33,34]. All subjects trunk, hip and dominant lower limb were stabilized with inelastic bands during the test.

Both isokinetic concentric knee extensor power, total work and isometric extensor torque were tested with the subjects in the sitting position, with the knees and hips flexed at 90° and in neutral hip adduction/ abduction and medial/lateral rotation. The rotational axis of the dynamometer was aligned to the lateral epicondyle of the femur and the lever arm attached three cm above the lateral malleolus.

The range of motion stipulated for the isokinetic test was from 90° to 20° of knee flexion and the angular speed was 60°.s^-1^. For the isometric test, the fixed position was 60° of knee flexion. For the isometric procedure, three series of five-second contractions with a 3-min interval between them were done after a submaximal series of familiarization. After a 10-min interval, the participants performed one isokinetic 1-minute series of maximal repetitions [33] after one submaximal familiarization separated by a 3-min period of recovery.

Standardized oral encouragement from a single evaluator was provided to stimulate the participants to produce their maximal effort during all tests. For statistical analyses, we used the highest absolute isometric peak torque normalized to BM, BMI and LW. Isokinetic power and total work, representing muscle endurance and the ability to generate force in a time-dependent manner [21], were represented by normalizing them to BM and LW. An acceptable test–retest reliability of knee torque measurements has been established [35].

### Statistical Analysis

The SPSS Statistics for Windows, Version 17.0 (SPSS Inc., Chicago, IL, USA) software package was used to analyze all data. The normality of distribution was verified by the Shapiro-Wilk test. The Student t-test and Mann-Whitney test were used according to parametric and nonparametric assumptions. Data were presented as mean ± SD for continuous variables and as median (minimum, maximum) for categorical variables. Pearson’s and Spearman’s coefficients were calculated to study associations by means of correlations between the main variables_._ The r values were interpreted using the following guidelines: 0.00 to 0.19 = none to slight, 0.20 to 0.39 = low, 0.40 to 0.69 = modest, 0.70 to 0.89 = high, and .90 to 1.00 = very high [36].

Stepwise multiple linear regression analysis was applied to predict VO_2peak_ achieved during CPX from 6MST results as well as from anthropometric and demographic variables. The Intraclass Correlation Coefficient (ICC) was used to verify reproducibility of the 6MST for the present sample. As the 6MST was reproducible in both conditions with no influence of procedure familiarization according to our ICC analysis, data of the second 6MST was used for the remaining analyzes.

A sample size of twenty-nine individuals was calculated using the GPower statistical package, Version 3.1.3 (Franz Faul Universität Kiel, Germany) according to the number of observations required for linear multiple regression analysis considering a 5% type I error, 85% power and 0.5 effect size.

A second sample size of 14 individuals was used to validate the predictive equation. A t-test was applied to test differences between the mean achieved VO_2peak_ at CPX and the equation-estimated value for both groups (i.e., LN and OB).

We had two missing values for strength measures, two for body composition, one for distribution, one for insulin resistance, one for hemodynamic measurements at the peak of CPX and none for ergospirometric metabolic and hemodynamic during both tests. All of the above were treated as missing data, not being replaced by an averaged value.

## Results

**Fig 1:**
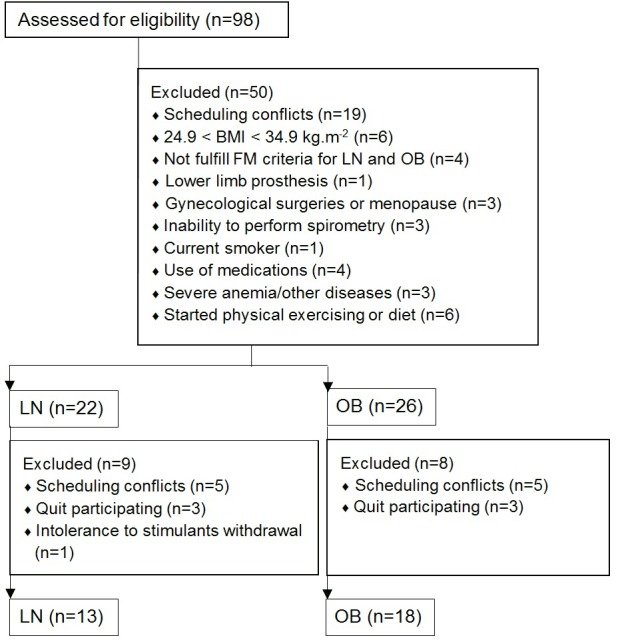
Flow diagram representing sample recruitment and loss. Recruitment, eligible subject’s assessment, sample loss and its causes and final studied sample. BMI, body mass index; FM, fat mass; LN, lean group; OB, obese group.

The cohort was recruited and evaluated from February 2013 to September 2014 and consisted of lean and obese young women. The flow diagram in Fig 1 demonstrates the recruitment, eligible subject assessment, sample loss and its causes and the sample achieved for final analyzes. Of the 98 potential study subjects assessed for eligibility, almost 32% of them were included in the final sample. The greatest sample loss occurred in the first phase of screening because we intended to obtain a more homogeneous sample in order to increase the internal validity of our findings.

All of the subjects included in the final cohort had normal lung function demonstrated by 94.1±9.0, 93.5±8.1 and 126.0±24.7 mean percentages of predicted forced expiratory volume in the first second (FEV_1_), forced vital capacity (FVC), and inspiratory capacity (IC), respectively.

The subjects were assessed by the Baecke Questionnaire and they had a mean absolute score of 5.5±1.8 and 6.2±1.4 of a maximum score of 15, a physical activity at work score of 2.1±0.7 and 2.3±0.7, a sport leisure time score of 1.8±0.5 and 1.9±0.5, and a physical activity leisure time score of 2.2±0.7 and 2.1±0.7 for LN and OB, respectively. As a result of this questionnaire, along with the 150 minutes per week physical activity threshold, all subjects were considered sedentary with little influence of physical activity level on our findings.

### Muscle mass, strength and power

Table 1 lists key characteristics of both cohorts. As expected, OB subjects had higher BF, abdominal fat distribution ratio, absolute muscle mass (LLM and ASMI), and absolute peak torque (PT). In contrast, the first-mentioned group presented the lowest values of percentage LM, strength and power normalized variables (Table 1).

**Table 1:** Anthropometric, demographic, body composition and muscle measures.

	LN (n = 13)	OB (n = 18)
***Anthropometric***		
Age (y)	32 ± 5	35 ± 5
Body Mass (kg)	57.2±5.0	112.6±22.8*
BMI (kg·m^-2^)	21.6±2.0	42.2±6.6*
***Body composition and distribution***		
BF (%)	29.1±3.3	47.9±3.3*
LM (%)	67.4±3.1	43.4±16.1*
Thigh circumference (cm)	52.5±3.2	72.5±9.3*
WHR	0.75±0.05	0.84±0.07*
***Muscle mass and function***		
ASMI (kg.m^-2^)	6.2±0.4	9.0±0.7*
LLM (kg)	6.5±0.3	9.1±0.9*
PT (N.m)	133.8±21.7	177.2±37.1*
PT/BM (N.m.kg^-1^)	2.3±0.7	1.6±0.4*
PT/LW (N.m.kg^-1^)	233.2±38.8	159.9±37.9*
PT/BMI (N.m.kg^-1^.m^2^)	6.2±0.9	4.3±0.9*
AVG Power/BM (W.kg^-1^)	1.0±0.2	0.7±0.2*
AVG Power/LW (W.kg^-1^)	5.7±1.0	4.0±1.0*
TW/BM (J.kg^-1^)	28.0±4.6	19.1±3.4*
TW/LW (J.kg^-1^)	164.9±27.4	116.1±25.6*

Data presented as mean ± SD. LN, lean group; OB, obese group; BMI, body mass index; BF, body fat mass; LM, lean body mass; WHR, Waist-to-hip ratio; ASMI, appendicular skeletal muscle mass index; LLM, appendicular lean mass of the dominant leg; PT, isometric extensor peak torque; BM, body mass; LW, leg weight; AVG, average; TW, extensor isokinetic total work.

* Intergroup differences between LN and OB, p<0.05.

In order to elucidate the metabolic profile of our studied sample, which possibly influences muscle function, Table 2 highlights the metabolic parameters of LN and OB according to their lipid, glycemic and insulin content as well as the representative index of insulin resistance. Except for age and low-density lipoprotein content, the groups were significantly different from each other with respect to these variables. Because we recognize metabolic profile, such as insulin sensitivity and resistance, as well as glucose and lipid metabolism, to be directly associated to muscle function as found in previous studies [37,38], a sub analysis of the obese group was performed, dividing groups according to an insulin resistance index using a 1.95 cutoff for Homeostasis Model Assessment (HOMA) [39] and NCEP ATP-III metabolic syndrome criteria [40]. According to these analyzes, neither the strength and muscular endurance variables during isometric and isokinetic tests nor the aerobic performance during maximal and functional exercise testing were influenced by metabolic profile because no difference were found between these subgroups.

**Table 2:** Metabolic characteristics.

	LN (n = 13)	OB (n = 18)
Fasting glucose (mmol/l)	4.8±0.4	5.1±0.4*
Fasting insulin (μu/ml)	6.4±2.3	14.4±7.1*
HOMA	1.46±0.51	3.28±1.60*
IR (n/n_group_)	0/13	7/13
Total cholesterol (mmol/l)	10.5±2.8	11.2±2.1*
HDL (mmol/l)	3.2±0.7	2.6±0.4*
LDL (mmol/l)	6.3±2.2	7.2±1.7
VLDL (mmol/l)	0.9±0.3	1.5±0.9*
Triglycerides (mmol/l)	4.6±1.4	7.5±4.5*

Data presented as mean ± SD. HOMA, homeostatic model assessment; IR, insulin resistant; HDL, high-density lipoprotein; LDL, low-density lipoprotein; VLDL, very low-density lipoprotein.

* Intergroup differences between LN and OB.

### Cardiorespiratory fitness and functional capacity

Metabolic, cardiovascular and perceptual variables from the CPX and 6MST, aside from performance and workload variables from the 6MST, are displayed in Table 3.

**Table 3:** Variables measured at peak exercise: CPX and 6MST.

	CPX		6MST	
	LN (n = 13)	OB (n = 9)	LN (n = 13)	OB(n = 9)
***Performance***				
NSC (n)	_	_	196±24	152±22*
Workload (W)	_	_	275.4±42.2	412.5±69.4*
***Metabolic data***				
VO_2peak_ (mL.kg^-1^.min^-1^)	32.4±4.2	21.2±3.1*	24.9±3.7^#^	17.6±3.1* ^#^
VO_2peak_ rel (%)	_	_	77±9	81±13
VO_2peak_/NSC (mL.kg^-1^.min^-1^.n)			0.13±0.01	0.12±0.04
RER	1.25±0.09	1.23±0.10	1.04±0.09^#^	1.07±0.10^#^
***Cardiovascular data***				
HR (bpm)	181±12	176±11	163±15^#^	159±15^#^
HR (%pred)	102±6	101±5	92±8^#^	90±9^#^
SBP (mmHg)	154±23	199±30*	140±13	179±25*
DBP (mmHg)	79±9	96±21*	67±12^#^	80±14* ^#^
***Simptons***				
Dyspnea	7 (5;10)	7.5 (3;10)	3 (0.5;8) ^#^	3.5 (0;10)^#^
Leg fatigue	4 (0;10)	3 (0;10)	3 (0;7)	2 (0;10)

Data are presented in mean±SD and median (min;max). CPX, cardiopulmonary exercise testing; 6MST, six-minute step test; LN, lean group; OB, obese group; NSC, number of step cycles; VO_2peak_, oxygen consumption at the peak of exercise; rel, oxygen consumption at 6MST relative to VO_2peak_ at CPX_;_ RER, respiratory exchange ratio; HR, heart rate; SBP, systolic blood pressure; DBP, diastolic blood pressure. Intergroup and intratest differences between

^*^LN and OB; and intragroup and inter-test differences between

^#^ CPX and 6MST.

As a measure of functional performance in our study, NSC, was higher in LN than OB and, inversely, workload (W) was higher in OB than in LN, as we expected, since the formula for calculating W takes into account individual BM and performance in the test, remaining fixed the constant value and the step height. The average NSC per minute in 6MST for LN and OB were 33±1 and 25±1, respectively.

**Fig 2:**
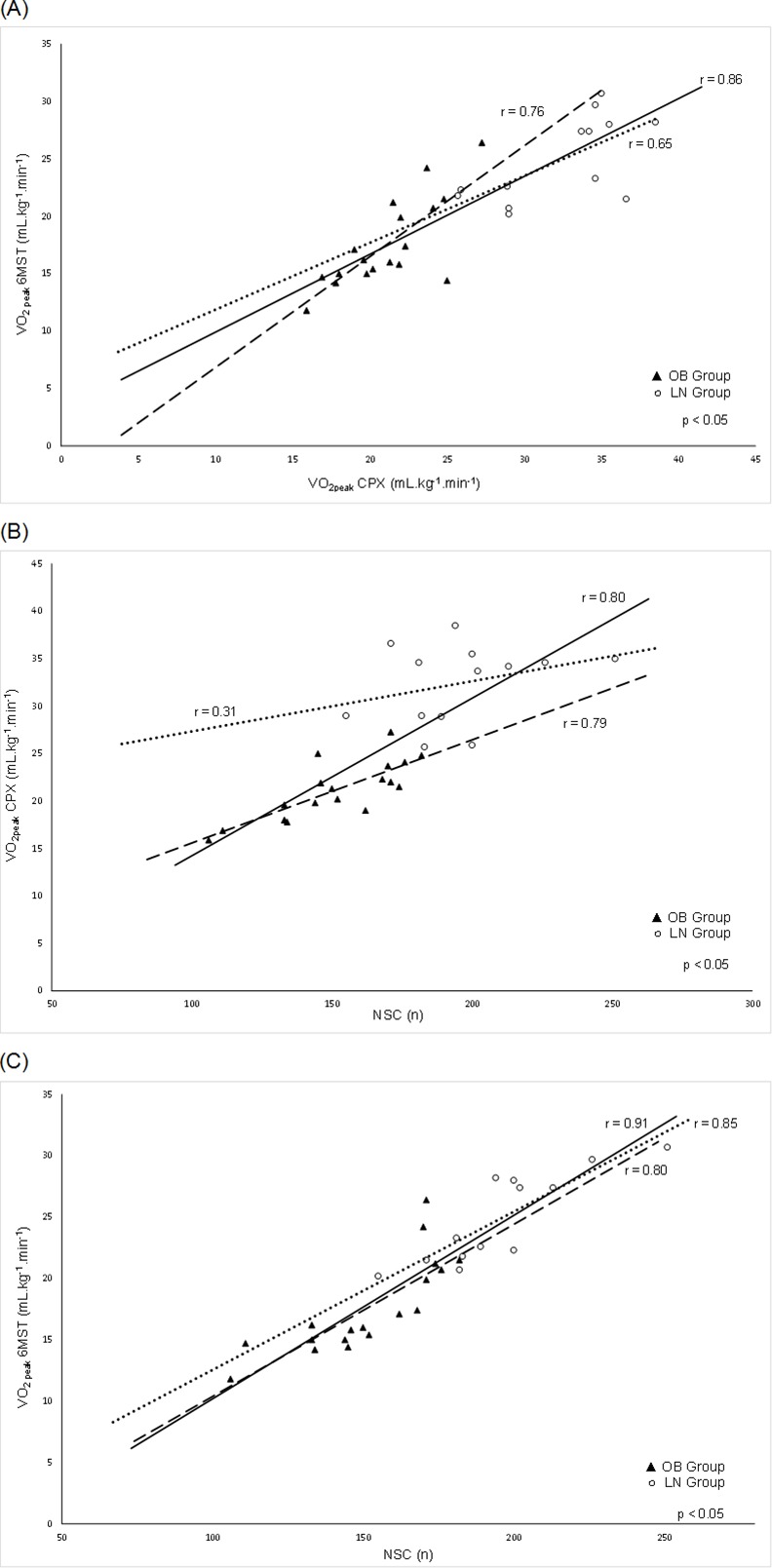
Correlations for LN, OB and overall sample for VO_2peak_ at CPX, VO_2peak_ and NSC at 6MST. Pearson’s coefficient correlations between cardiorespiratory fitness and functional main variables between (A) VO_2peak_ at CPX and 6MST; (B) VO_2peak_ at CPX and NSC; (C) VO_2peak_ at 6MST and NSC. VO_2peak,_ oxygen consumption at the peak of exercise; CPX, cardiopulmonary exercise testing; 6MST, six-minute step test; NSC, number of step cycles; OB, obese group; LN, lean group. Dashed line: OB; dotted line: LN; continuous line: LN and OB (overall sample).

Strong overall correlations between CPX and 6MST main variables were found, as we can see in Fig 2A and 2B. As a result, we have investigated the 6MST performance ability to predict VO_2peak_. Additionally, we observed in the same figure (Fig 2C) that NSC was strongly correlated with VO_2peak_ reached in this functional test.

According to the averaged-single measures ICC analysis (0.97; 95%CI: 0.898–0.993; p<0.001), NSC in the 6MST was reproducible, as previously demonstrated in other populations [12,13].

### Predictive equation and validity assessment

The univariate analysis demonstrated that VO_2peak_ at CPX correlated significantly with BMI (r = -0.84; p<0.001), NSC (Fig 2B), and age (r = -0.40; p<0.05) and were subsequently inserted into a stepwise multiple linear regression analysis to investigate the ability of these easily accessible variables to predict VO_2peak_. Taking all predictors together, the final model explained 83% of the total variance in VO_2peak_ during CPX. The reference equation obtained was: VO_2peak_ = 35.335 - (0.328 x BMI) + (0.069 x NSC)–(0.298 x age), with a standard error of the estimate = 2.9 mL·kg^-1^·min^-1^ (Durbin-Watson test = 2.2; p<0.01).

To assess usefulness of the purposed functional test to predict the gold standard measure, validity of the equation was tested by means of data from another sample of 14 subjects (seven lean and obese women) evaluated in a previous pilot study. Their characteristics were: age 36 ± 9 years old; height, 1.65 ± 0.1; BM, 60.4 ± 7.3 kg; and BMI, 22.1 ± 1.8 kg·m^-2^ for lean women and age 32 ± 5 years old; height, 1.62 ± 0.1; BM, 118.0 ± 23.8 kg; and BMI, 45.6 ± 12.1 kg·m^-2^ for obese women. There were no differences between the mean VO_2peak_ registered during the performed test and the VO_2peak_ calculated by the equation: 28.0 ± 5.0 *vs*. 30.4 ± 3.5 mL·kg^-1^·min^-1^ for LN and 23.3 ± 4.9 *vs*. 21.0 ± 6.2 mL·kg^-1^·min^-1^ for OB (p>0.05).

### Association between muscle function, cardiorespiratory fitness and functional capacity

In order to investigate to what extent muscle function accounts for physical performance during the 6MST and during the CPX, associations between CRF, functional capacity during the 6MST and knee extensor strength, power and total work are shown in Table 4. The relationship between body composition and performance variables is also listed.

**Table 4:** Correlation coefficients between VO_2peak_ at CPX, VO_2peak_ at 6MST, 6MST performance (NSC) and body composition and muscle function variables.

	VO_2peak_ CPX	VO_2peak_ 6MST	NSC	p-value
BF (%)	-0.74^a^	-0.57 ^a^	-0.58 ^a^	<0.001
LM (%)	0.70	0.54	0.62	<0.05
PT/BMI (N.m.kg^-1^.m^2^)	0.74	0.64	0.67	<0.001
PT/BM (N.m.kg^-1^)	0.77	0.69	0.71	<0.001
PT/LW (N.m.kg^-1^)	0.76	0.68	0.72	<0.001
AVG power/BM (W.kg^-1^)	0.54	0.54	0.50	<0.001
AVG power/LW (W.kg^-1^)	0.58	0.52	0.60	<0.001
TW/BM (J.kg^-1^)	0.75	0.70	0.73	<0.001
TW/LW (J.kg^-1^)	0.67	0.67	0.69	<0.001

VO_2peak_, oxygen consumption at the peak of exercise, in mL.kg^-1^.min^-1^; CPX, cardiopulmonary exercise testing; 6MST, six-minute step test; NSC, number of step cycles (n); BMI, body mass index; BF, body fat mass; LM, lean body mass; WHR, PT, isometric extensor peak torque; BM, body mass; LW, leg weight; AVG, average; TW, extensor isokinetic total work.

^a^Spearman’s coefficient correlation.

According to these results, the easily assessed performance variable (i.e., NSC in the 6MST) well represents the VO_2peak_ in the same test regarding their association with muscle strength and power. In addition, the 6MST performance seems to be also consistent with the CPX regarding their association with muscle function.

## Discussion

To our knowledge, this is the first study to compare and correlate the cardiorespiratory responses between the cadence-free six-minute step test with the gold standard exercise testing in lean and obese young women. Primary findings from our study are: 1) accurate predictive equation could be developed for both groups from the 6MST; and 2) measures of muscle strength and power were significantly associated with aerobic capacity and functional capacity.

These results suggest high internal validity of our findings, as the study used well-validated measures [3–4,13,41–44] for both muscle strength, power and CRF assessment. Therefore, our results allow us to highlight the usability and applicability of these findings in lean and obese young women, for which a wide range of cardiorespiratory responses and functional performance are observed.

### Muscle mass, strength and power

Although the OB presented with more absolute muscle mass, all normalized variables expressing strength and power were markedly reduced in this group as previously demonstrated in other studies [19,20]. Maffiuletti et al. (2007) [19] demonstrated that quadriceps femoris muscle fatigue during maximal voluntary contractions was significantly greater in obese men (grades II and III) and relative torque and power values were ≈32% lower in obese compared to non-obese subjects. Paolillo et al. (2012) [21], evaluating postmenopausal obese women, found lower peak torque in obese compared to normal-weight and overweight in accordance with the previous study. However, they found an equivalent power and total work as well as lower quadriceps fatigability in obese compared to non-obese groups, expressed by peak torque decline over a 1-minute protocol in a higher angular velocity endurance test than that adopted in our study. The reduced fatigability in their study might be explained by an aging weight-bearing effect as an adaptability mechanism to support the high load imposed by their own body mass throughout their usual daily activities. By mimicking a constant exercise load, it could promote fatigue-resistant effects by increasing slow-twitch motor units as opposed to fast-twitch motor units, preserving their compromised muscle strength, but enhancing their endurance capacity [21,45,46].

Surprisingly, only a limited number of researchers have investigated these muscular features in obese adults, especially in obese women. However, according to those previously mentioned, it is well accepted that obese adults generate higher absolute strength and power with the lower extremity musculature than their lean counterparts, likely due to the larger amount of lean mass. Thus, it is preferable to express muscle performance in terms of body mass, to respect the specificity of the contractions performed during daily activities [47], considering that the majority of them require body mass lifting or support. Therefore, the lower quadriceps muscle torque and power relative to BM observed in obese subjects could be considered as major contributors to the reduced functional capacity in obese individuals.

In this sense, normalization per BM, instead of the more common normalization per LLM, was chosen to represent in some way the load bearing on the muscles, which is essentially the major biomechanical constraint in obese subjects, therefore representing their functional impairment with regard to their whole body load [48]. Apart from that, all comparisons between groups regarding strength, power and total work measures normalized by LLM were below statistical significance in our study. Other investigations reported similar findings in obese adolescents, which allow us to speculate that quadriceps force-generating capacity depends upon many neural and muscular factors, such as the extent of motor unit activation and muscle cross-sectional area, rather than muscle mass solely [22].

Furthermore, it must not be overlooked that although having strong concurrent validity with the gold standard [49], our method of evaluating body composition likely underestimated intermuscular adipose tissue, which has an important negative influence on muscular strength and resistance by promoting a chronic proinflammatory status due to deleterious cytokines release. Besides affecting its function by means of muscular or systemic stimulus, this abnormal infiltration seems to be related to metabolism impairment and mechanical disadvantage for muscle fiber contraction [50]. In this sense, Hilton et al. (2008) [51] observed an inverse correlation between muscle power, strength and physical performance scores and fat infiltration.

Particularly for our studied sample, it is possible that at an early stage of life these metabolic changes may not yet reflect muscle force worsening, but it could be observed after long term exposure of the unhealthy metabolic condition. This has been confirmed by Karelis et. al., (2007) [52] who found higher levels of insulin sensitivity were associated with greatest values of lower-body muscle strength by means of a 1- repetition maximum test in overweight and obese sedentary postmenopausal women. Another suggested factor could be the cardiovascular properties of estrogen in adult women as an important factor accounting for the hormonal cardioprotective effect, such as vasomotor and antioxidant effects, even in the presence of abnormal lipid content [53].

In this context, muscle force-generating ability should be affected by a range of worsening conditions, to varying degrees, compromising a better performance in daily living activities such as transitioning from sit to stand, climbing stairs, getting up from squatting, walking, and others. Many authors have demonstrated that individuals with higher muscle strength scored better in physical performance in a variety of activities than their low-strength counterparts [16,18,54].

### Cardiorespiratory fitness and functional capacity

It is particularly notable that CPX elicited higher metabolic and cardiovascular responses compared to the 6MST in both LN and OB. This fact reinforces the submaximal or near maximal profile of functional step testing in which both groups reached approximately 91% of the predicted heart rate, differing from that achieved in the CPX, and approximately 79% of the VO_2peak_ obtained during the CPX. It is also important to note that the ratio of VO_2peak_ achieved during the 6MST by its NSC was not different between groups, which explains the equivalent intensity during this functional test for both groups, corroborating their percentage of predicted HR achieved in the 6MST. Despite the discrepancy between maximal and submaximal exercise testings, both groups had lower perceived responses during the 6MST compared to the CPX only with respect to dyspnea. This may reflect the fact that the 6MST entails a type of lower-limb physical perceived exertion similar to that during the CPX.

Despite having a self-paced profile, the 6MST might be defined as a constant-load test observed by the almost equal NSC throughout each minute in contrast to the incremental behavior of the CPX. Although each protocol differs from one another, the correlations found between their main variables strengthens the possibility of the 6MST use in clinical settings as a further alternative to assess CRF.

In relation to Fig 2A and 2B expressing the VO_2peak_ at the maximal and the VO_2peak_ at submaximal test as well as 6MST performance correlations_,_ it is worth mentioning that the values reached by the LN (white circles) were all proportionately higher than for the OB (black triangles). Additionally, it is important to note that the proximity of the black triangles suggests a more homogeneous exercise response in obese women than in non-obese, which are more dispersed in the above-mentioned figures. It seems that the main common feature among obese women, namely the greatest BMI, provided the higher homogeneity regarding their CRF pattern compared to LN.

Many authors have focused on developing predictive equations from submaximal tests or their ability to accurately identify CRF in healthy and disabled population [5–11,14,15]. Neves et al. (2015) [7] found that maximal cardiorespiratory responses in the shuttle walk test agreed with those obtained in the CPX, and the developed equation showed viability for the prediction of VO_2peak_ in healthy sedentary men. Moreover, as in our study, BMI and the functional test performance, on that occasion the gait speed, explained 40.6% (p = 0.001) of the variance in VO_2peak_. Baillot et al. (2015) [55], in turn, found that a physical functional capacity score, age, quadriceps strength and BMI explained 57% of the six-minute walking test distance variance in obese individuals.

### Association between muscle strength and power, cardiorespiratory fitness and functional capacity

To the best of our knowledge, there is no evidence-based studies that have applied a single functional test in normal-weight and obese sedentary young women with the same predictive purpose. Nor do we know about the relationship between the performance in the step test and muscle function in this population. Because of the 6MST powerful ability to predict CRF, we intended to investigate what mechanisms would influence its varied performance in lean and obese women. In this sense, we found moderate to strong correlations between both VO_2peak_ and NSC at 6MST and muscle strength and power variables. It becomes clear from our results that the obese young women had lower quadriceps strength and power as well as a lower performance in 6MST, which seems to explain, beyond CRF, their limited ability to perform this daily task. Corroborating these findings, Hulen et al. (2002) [23] also detected correlations between isokinetic strength measurements and VO_2peak_ in young obese women. Barbat-Artigas et al. (2014; 2013), by evaluating elderly individuals, showed that obesity and age influenced the relationship between knee extension strength and physical function, which were correlated with each other. Regarding physical function, the authors took into account a composite score of daily activity tasks, including a time-limited step test.

Others found the average power per BM to be correlated with individual’s performance during predominantly anaerobic tasks under maximal effort, such as in stair climbing and other activities requiring vertical displacement [25]. Some studies have found that higher BF composition and BMI result in a power decline in obese individuals, especially in women. Therefore, obesity overload seems to strongly influence leg muscle performance throughout a high-intensity task [19,24].

### Clinical applicability

The results of our study have primary importance by adding to the scope of knowledge regarding readily available methods, in this case the 6MST, to estimate the gold standard measure of CRF without the need of a highly specialized team and costly equipment. In this context, future research should further assess the usefulness and predictive ability in other populations, further expanding clinical applications.

### Study limitations

Our findings could not be extrapolated to male individuals as well as to elderly women. Another limitation is that body composition method analysis used in our study may have underestimated fat mass and/or overestimated lean mass percentage in our sample.

## Conclusion

In conclusion, CRF, functional capacity assessed by the 6MST as well as quadriceps muscle strength and power were lower in obese women compared to lean young women. Moreover, these outcomes are directly associated to each other and inversely related to BF, BMI, and age. Our findings indicate that 6MST performance, well represented by its NSC, BMI, and age, can predict VO_2peak_ assessed by the gold standard CPX, therefore enhancing the usefulness of these readily accessible variables in primary-care settings and rehabilitation clinics as part of the physical screening routine.
